# Primary Central Nervous System Lymphoma: A Case Report of the Neurological Great Mimicker

**DOI:** 10.7759/cureus.97478

**Published:** 2025-11-21

**Authors:** Sara Costa, Maria Carlos Pereira, Rui Felgueiras, Francisco Almeida, Gustavo Barbosa, Margarida Calejo, Ricardo Taipa, Diogo Costa

**Affiliations:** 1 Department of Neurology, Unidade Local de Saúde de Santo António, Porto, PRT; 2 Department of Neuroradiology, Unidade Local de Saúde de Santo António, Porto, PRT; 3 Department of Intensive Care, Unidade Local de Saúde de Santo António, Porto, PRT; 4 Department of Neurology, Unidade Local de Saúde de Matosinhos, Matosinhos, PRT; 5 Department of Neuropathology, Unidade Local de Saúde de Santo António, Porto, PRT

**Keywords:** brain biopsy, corticosteroid therapy, diagnostic challenges, diffuse large b-cell lymphoma, primary central nervous system lymphoma, rapidly progressive dementia

## Abstract

Primary central nervous system lymphoma (PCNSL) is a rare lymphoproliferative disorder that primarily affects immunocompromised individuals. It presents with diverse neurological phenotypes, making its diagnosis challenging and frequently delayed. Clinical heterogeneity is compounded by the limitations of available diagnostic methods, and the use of corticosteroids can mask imaging and histological characteristics, further increasing diagnostic delay. We describe a case of a 68-year-old woman with a history of Hashimoto’s thyroiditis who presented with rapidly progressive dementia (RPD). Initial testing revealed positive anti-thyroglobulin antibodies, leading to a suspicion of steroid-responsive encephalopathy associated with autoimmune thyroiditis. She was treated with corticosteroids, without clinical improvement. Brain magnetic resonance imaging (MRI) showed supratentorial hyperintense lesions on T2-weighted fluid-attenuated inversion recovery (T2/FLAIR) sequences without diffusion restriction. Additionally, a nodular cerebellar lesion exhibited contrast enhancement and restricted diffusion. Cerebrospinal fluid (CSF) analysis, including cytology and flow cytometry, was unremarkable. The first brain biopsy performed four months after corticotherapy was inconclusive, revealing a non-specific inflammatory pattern. Only a second brain biopsy, performed nine months after corticosteroid therapy, confirmed the diagnosis of PCNSL.

## Introduction

Rapidly progressive dementias (RPDs) constitute a broad spectrum of diseases where cognitive decline evolves to dementia or death faster than expected within one to two years [1]. It encompasses a heterogeneous differential diagnosis, including immune-mediated conditions (e.g., autoimmune encephalitis, steroid-responsive encephalopathy associated with autoimmune thyroiditis (SREAT)), infectious conditions, toxic-metabolic disorders, neoplastic conditions (both primary and secondary central nervous system (CNS) tumors), prion diseases, and atypical presentations of common neurodegenerative disorders [1].

Primary central nervous system lymphoma (PCNSL) is a rare, aggressive extranodal non-Hodgkin lymphoma confined to the CNS. It predominantly affects immunocompromised patients but can also occur in immunocompetent individuals, with a median age of onset of 66 years [2]. Clinically, it can have diverse neurological presentations depending on the affected brain structure. Imaging features on magnetic resonance imaging (MRI) and cerebrospinal fluid (CSF) findings are often non-specific. Cytology and flow cytometry of CSF have limited sensitivity, making a brain biopsy often necessary for definitive diagnosis [3].

This report describes a case of rapidly progressive dementia caused by PCNSL, highlighting the diagnostic challenges associated with this condition.

## Case presentation

**Figure 1:**
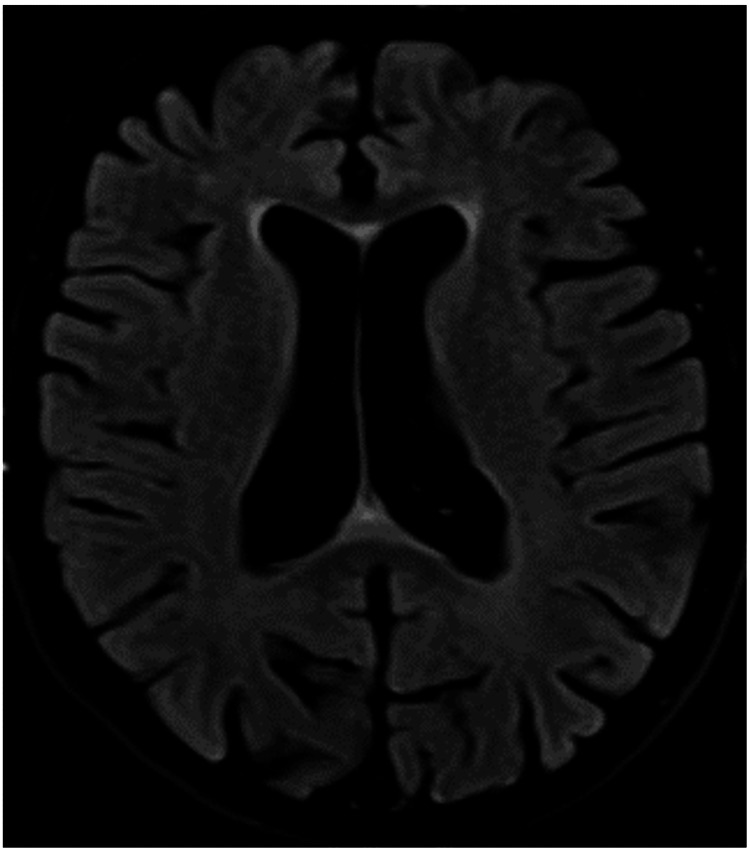
Brain MRI findings: Axial FLAIR image obtained three months after clinical onset. MRI, magnetic resonance imaging; FLAIR, fluid-attenuated inversion recovery

A 68-year-old, immunocompetent woman, independent in her activities of daily living (ADLs) (modified Rankin Scale (mRS) score 0), with a history of Hashimoto’s thyroiditis, hypertension, dyslipidemia, and deep vein thrombosis, presented with a two-month history of personality changes and apathy [4]. She remained independent during the initial phase of her illness. One month later, she experienced a transient loss of consciousness, from which she recovered within minutes, but had no memory of the event. No involuntary movements, sphincter incontinence, or tongue biting were noted. Following this episode, her apathy worsened, and she developed a gait characterized by small shuffling steps. She was first evaluated by a neurologist at this time, three months after the onset of symptoms. An initial brain MRI (Figure 1) showed no significant abnormalities, and she was prescribed lamotrigine (100 mg twice daily) for a presumed epileptic seizure.

Four months later, she experienced another episode of transient loss of consciousness, and her cognitive decline progressed more rapidly over the following month. She became fully dependent in ADLs due to worsening apathy, socially inappropriate behavior, disorganized speech, and visuospatial disorientation. Additionally, her gait impairment worsened, with the patient only being able to walk with unilateral support.

Eight months after the first symptoms, she was admitted as an inpatient for further evaluation. CSF analysis revealed a normal white blood cell (WBC) count (1 cell/μL), normal protein levels (0.3 g/L), and normal glucose levels (3 mmol/L), with negative bacterial, viral, and fungal studies. Hematology, erythrocyte sedimentation rate, renal function, and liver function tests were normal. Serologic tests for hepatitis B, C, HIV, *Treponema pallidum,* and *Borrelia *were negative. Autoimmune screening, including complement levels (C3, C4), anti-nuclear antibodies (ANA), anti-double-stranded DNA (anti-dsDNA), anti-neutrophil cytoplasmic antibodies (ANCA), anti-Sjögren's syndrome type A (anti-SSA) and type B (anti-SSB) autoantibodies, anti-gliadin, and anti-endomysial antibodies, was negative. Anti-neuronal antibodies were also negative in blood and CSF (Table 1). Thyroid function tests (thyroid-stimulating hormone (TSH), free thyroxine (T4), and triiodothyronine (T3)) were normal, anti-thyroid peroxidase antibodies were negative, but anti-thyroglobulin antibodies were elevated (>115 U/mL). High-dose intravenous methylprednisolone (1 g for five days) followed by oral prednisolone (1 mg/kg/day for three months) was administered, but no clinical improvement was observed.

**Table 1:** Anti-neuronal antibodies tested in CSF and blood. CSF, cerebrospinal fluid; AMPAR, 2-amino-3-(5-methyl-3-oxo-1,2-oxazol-4-yl) propanoic acid receptor; ANNA, antineuronal nuclear antibody; CASPR2, contactin-associated protein-like 2; CV2/CRMP5, collapsin response-mediator protein 5; DNER, delta/notch-like epidermal growth factor-related receptor; DPPX, dipeptidyl-peptidase-like protein 6 encephalitis; GABA, γ-aminobutyric acid; GAD, glutamic acid decarboxylase; LGI1, leucine-rich glioma-inactivated 1; NMDAR, N-methyl-D-aspartate receptor; PCA, Purkinje cell cytoplasmic antigen; SOX, anti-Sry-like high mobility group box; Zic4, zinc finger protein 4

Anti-neuronal antibodies	Blood	CSF
Anti-Yo (PCA-1)	Negative	-
Anti-Hu (ANNA-1)	Negative	-
Anti-Ri (ANNA-2)	Negative	-
Anti-Tr (DNER)	Negative	-
Anti-GAD65	Negative	-
Anti-Zic4	Negative	-
Anti-titin	Negative	-
Anti-SOX1	Negative	-
Anti-recoverin	Negative	-
Anti-Ma2	Negative	-
Anti-CV2/CRMP25	Negative	-
Anti-amphiphysin	Negative	-
Anti-NMDAR	Negative	Negative
Anti-potassium channel: anti-LGI1, anti-CASPR2	Negative	Negative
Anti-AMPAR	Negative	Negative
Anti-recetor GABA_b_R	Negative	Negative
Anti-DPPX	Negative	Negative

Twelve months after initial symptoms and one month after completing corticosteroid therapy, the patient’s neurological state kept worsening, leading to readmission for further evaluation.

**Figure 2:**
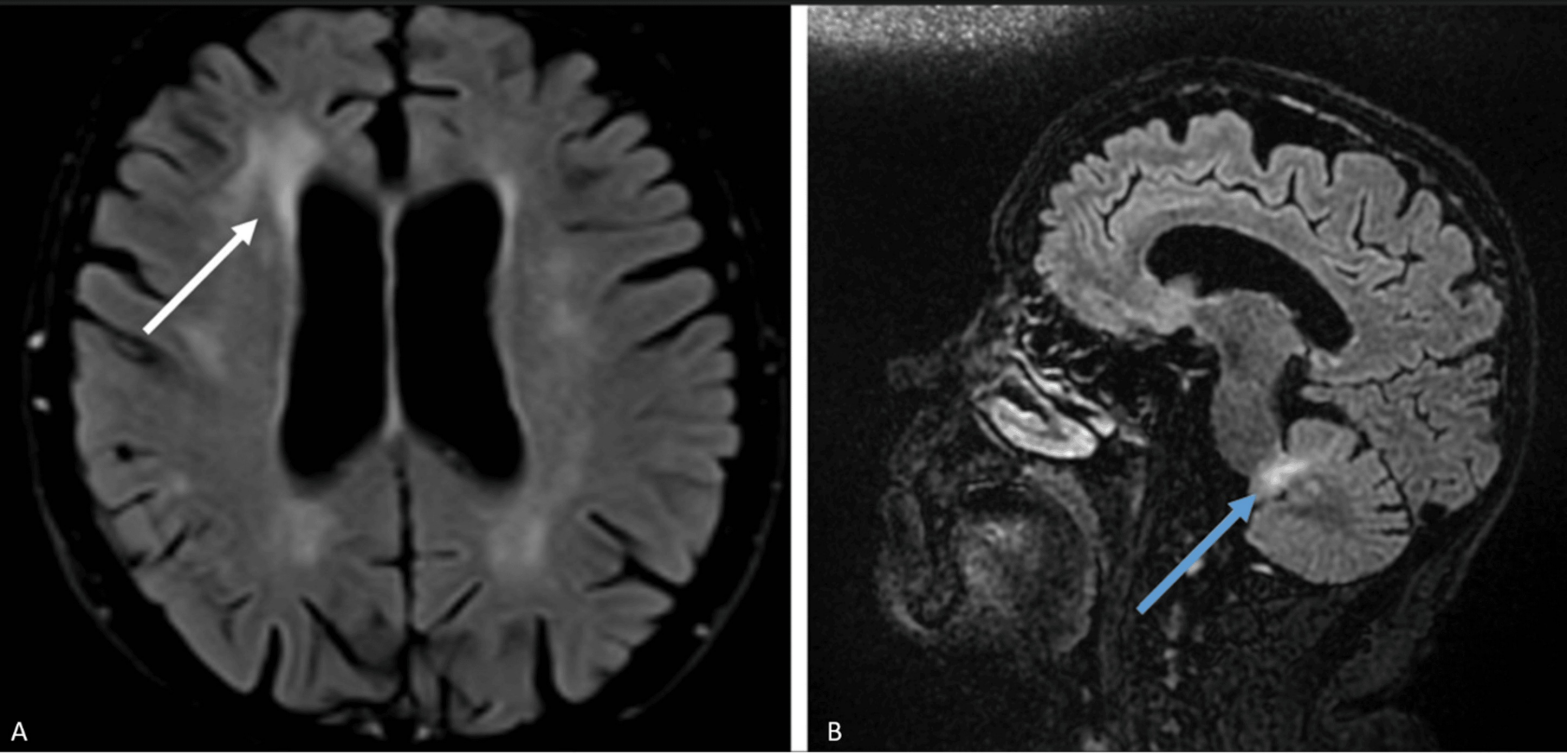
Brain MRI findings one month after corticosteroid therapy: Axial (A) and sagittal (B) FLAIR images show marked dilation of the lateral ventricles, diffuse periventricular and subcortical hyperintense lesions (white arrow), and a focal cerebellar hyperintense lesion (blue arrow). MRI, magnetic resonance imaging; FLAIR, fluid-attenuated inversion recovery

The neurological examination revealed psychomotor slowing, positive glabellar reflex, non-fluent speech, ideomotor apraxia, mild facial hypomimia, action tremor of the upper limbs, mild bradykinesia of the upper limbs, and generalized oppositional paratonia. The gait was characterized by small steps, with postural instability in the standing position, only possible with unilateral support. A second brain MRI (Figure 2) showed new T2-weighted fluid-attenuated inversion recovery (T2/FLAIR) hyperintense supratentorial white matter lesions, without contrast enhancement or diffusion restriction, and a cerebellar lesion with slight contrast enhancement. A second lumbar puncture was performed, including 14-3-3 protein analysis, which was negative, and cytology and flow cytometry did not detect neoplastic cells in the CSF. The measurement of biomarkers for Alzheimer's disease, including total tau (t-tau), phosphorylated tau (p-tau), amyloid-beta (Aβ) 42, and the Aβ42/40 ratio, was unremarkable. Systemic and anti-neuronal autoimmune studies were repeated and remained negative. 

**Figure 3:**
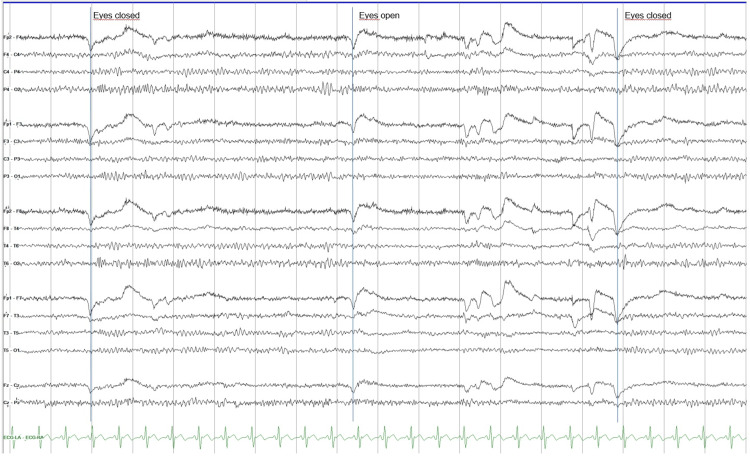
Posterior dominant rhythm at a frequency of 10-11 Hz, medium amplitude (30-60 µV), symmetrical, and reactive to eye opening. No epileptiform discharges or abnormal slow activity are observed.

An electroencephalogram (Figure 3) was performed, showing no paroxysmal activity or pathological slow activity. 

**Figure 4:**
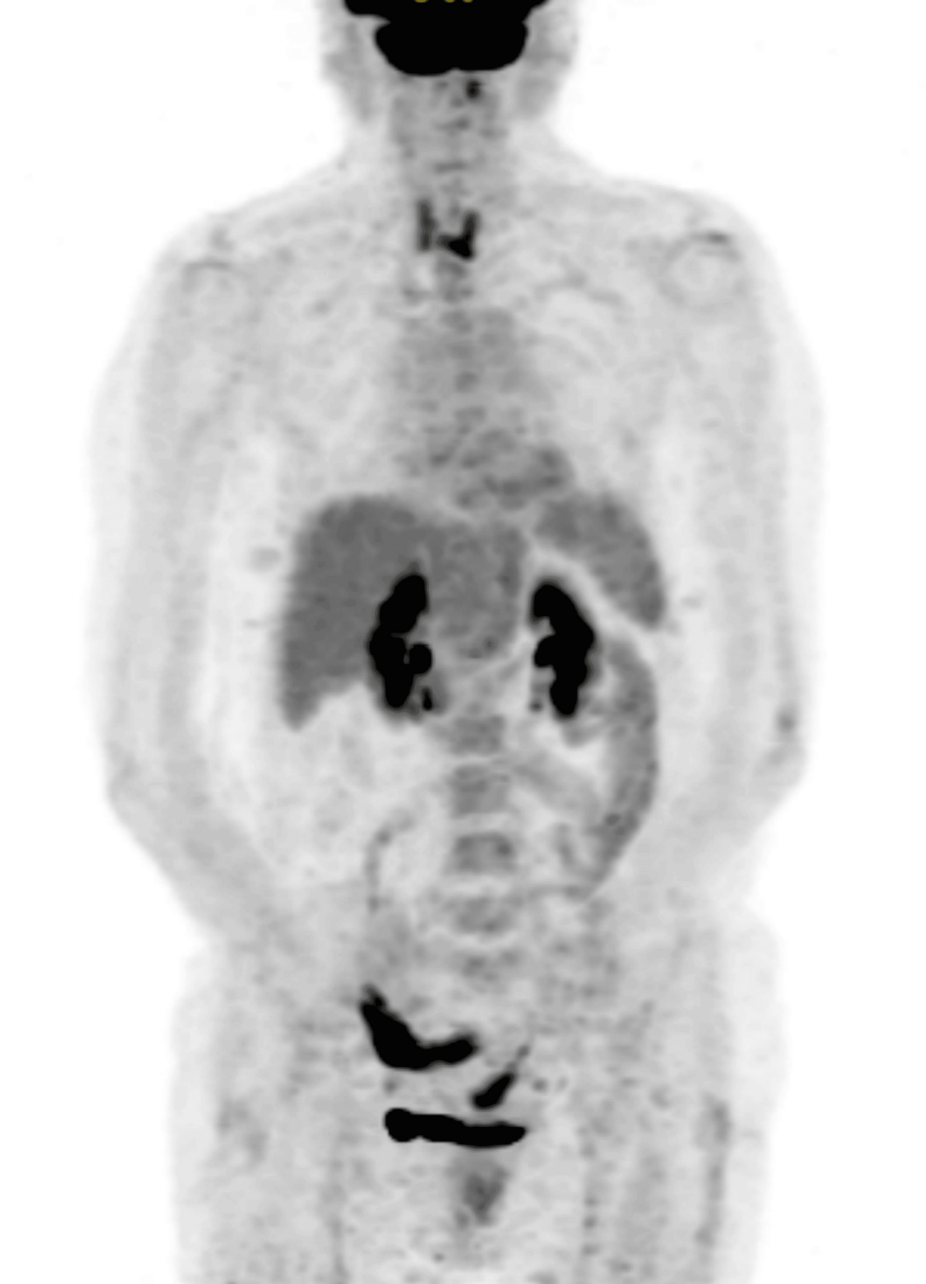
F-18 FDG PET/CT findings: No hypermetabolic lesions suggestive of active neoplastic disease are identified. Diffuse thyroid uptake is consistent with known Hashimoto’s thyroiditis. F-18, fluorine-18; FDG, fluorodeoxyglucose; PET/CT, positron emission tomography/computed tomography

The whole-body positron emission tomography/computed tomography (PET/CT) (Figure 4) revealed no hypermetabolic alterations suggestive of neoplastic lesions. The neuropsychological evaluation demonstrated a generalized cognitive decline with predominant impairment of short-term and visual memory and executive functions.

**Figure 5:**
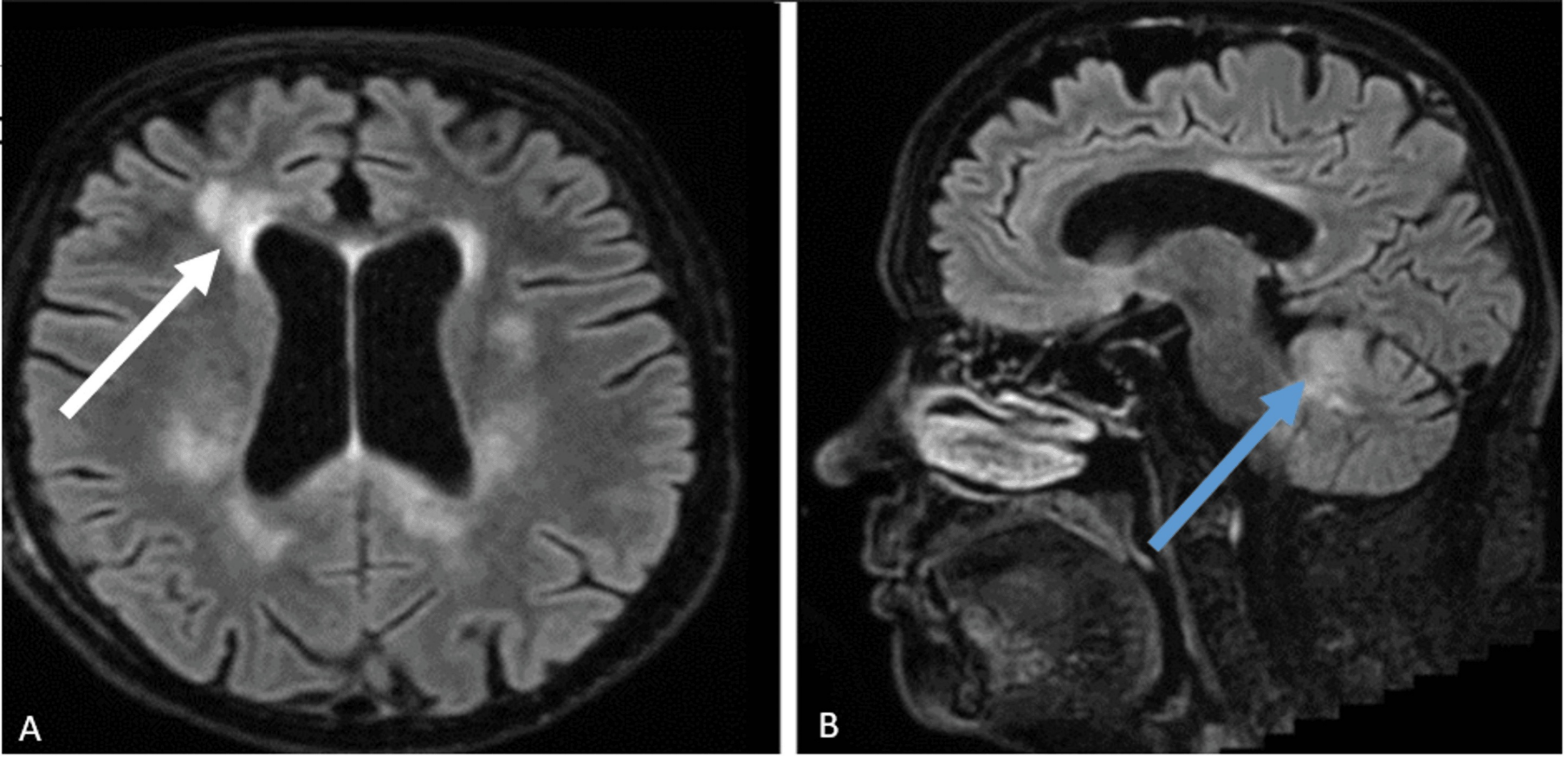
Brain MRI findings three months after corticosteroid therapy: Axial (A) and sagittal (B) FLAIR images demonstrate a hyperintense cortico-subcortical lesion in the right frontal lobe (white arrow) and a hyperintense lesion in the cerebellum (blue arrow). MRI, magnetic resonance imaging; FLAIR, fluid-attenuated inversion recovery

Fourteen months after the clinical onset and three months after corticotherapy, the patient continued to experience cognitive decline, with no clinical response to immunomodulatory treatment. Follow-up brain MRI (Figure 5) revealed additional supratentorial white matter lesions along fiber tracts, some fluctuating over time, along with a right frontal lesion without contrast enhancement, and the cerebellar lesion showed contrast enhancement without diffusion restriction.

Fifteen months after symptom onset and four months after corticosteroid treatment, due to radiological worsening, lack of clinical response, and the absence of a definitive diagnosis, the patient was referred for a stereotactic biopsy of the right frontal lesion.

**Figure 6:**
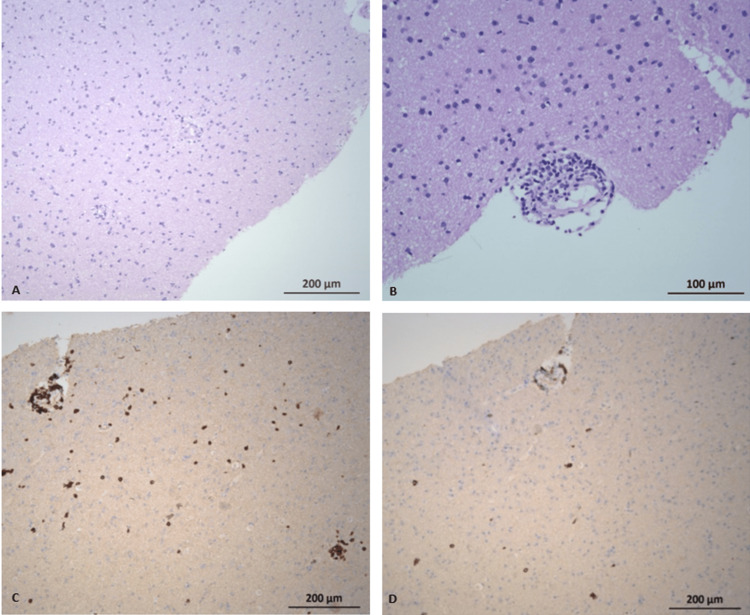
(A, B) H&E: Increased cellularity, particularly in the white matter, with small perivascular inflammatory infiltrates and no evidence of vasculitis. (C, D) Immunohistochemistry (CD3 and CD20): Perivascular inflammatory infiltrates are present. H&E, hematoxylin and eosin; CD: cluster of differentiation

Histopathology revealed T-cell-predominant inflammatory infiltration of white matter, with no evidence of vasculitis (Figure 6). At this point, a diagnosis of autoimmune encephalitis was considered. The patient was started on treatment with rituximab 1 g, of which she completed one cycle (days 1 and 15).

**Figure 7:**
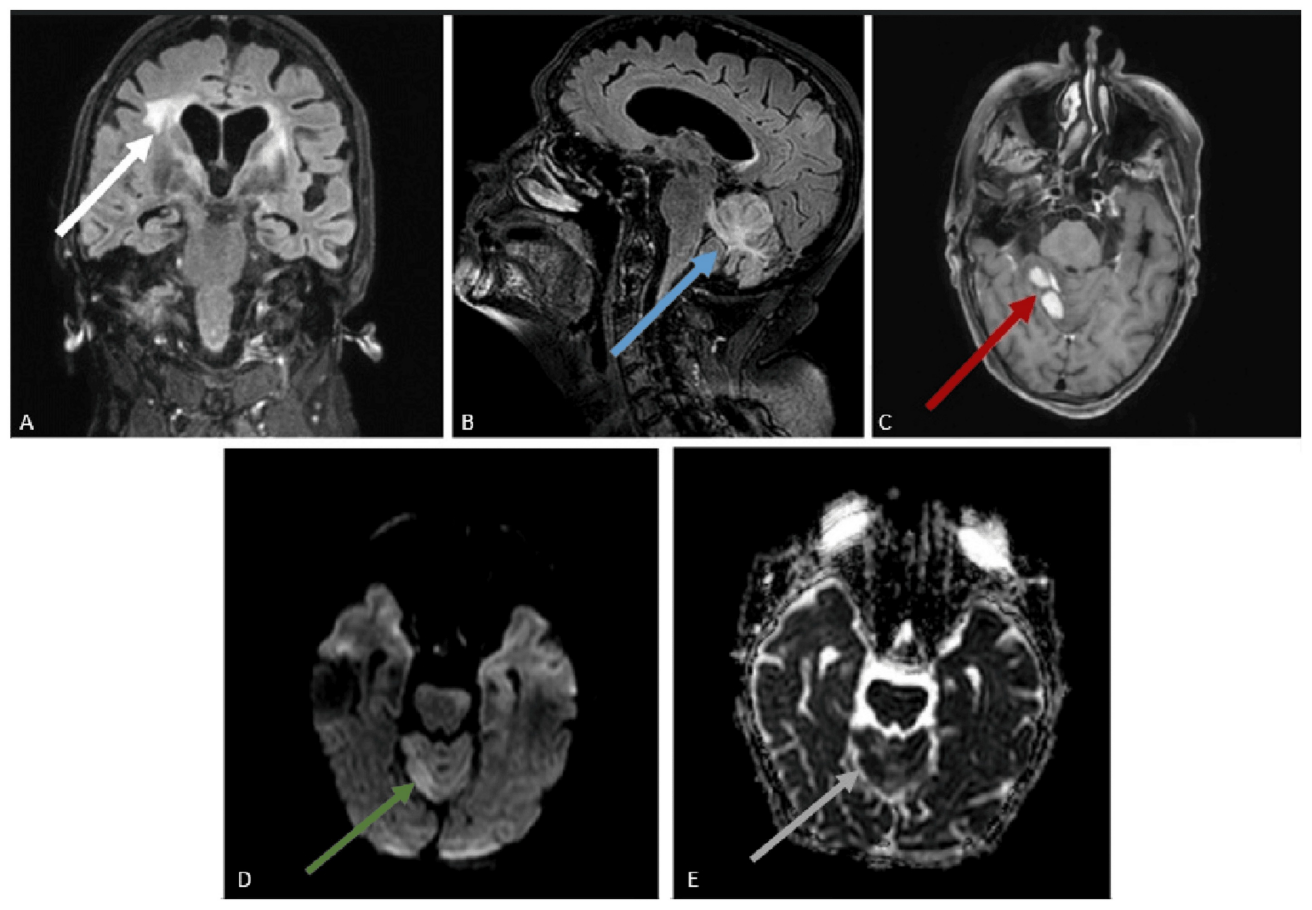
Brain MRI findings eight months after corticosteroid therapy: Coronal (A) and sagittal (B) FLAIR images show cortical and subcortical hyperintense lesions in the periventricular white matter (white arrow) and cerebellum (blue arrow). A T1-weighted sequence with gadolinium contrast (C) demonstrates a homogeneously enhancing cerebellar lesion (red arrow). Diffusion-weighted imaging (D) reveals restricted diffusion within the cerebellar lesion (green arrow), confirmed by corresponding low signal intensity on the apparent diffusion coefficient map (grey arrow) (E). MRI, magnetic resonance imaging; FLAIR, fluid-attenuated inversion recovery

No clinical improvement was noted three months after rituximab treatment. Another brain MRI (Figure 7) was performed. The imaging showed disease progression, with new T2/FLAIR hyperintense lesions throughout the white matter, without diffusion restriction, as well as a cerebellar lesion with diffusion restriction and contrast enhancement.

**Figure 8:**
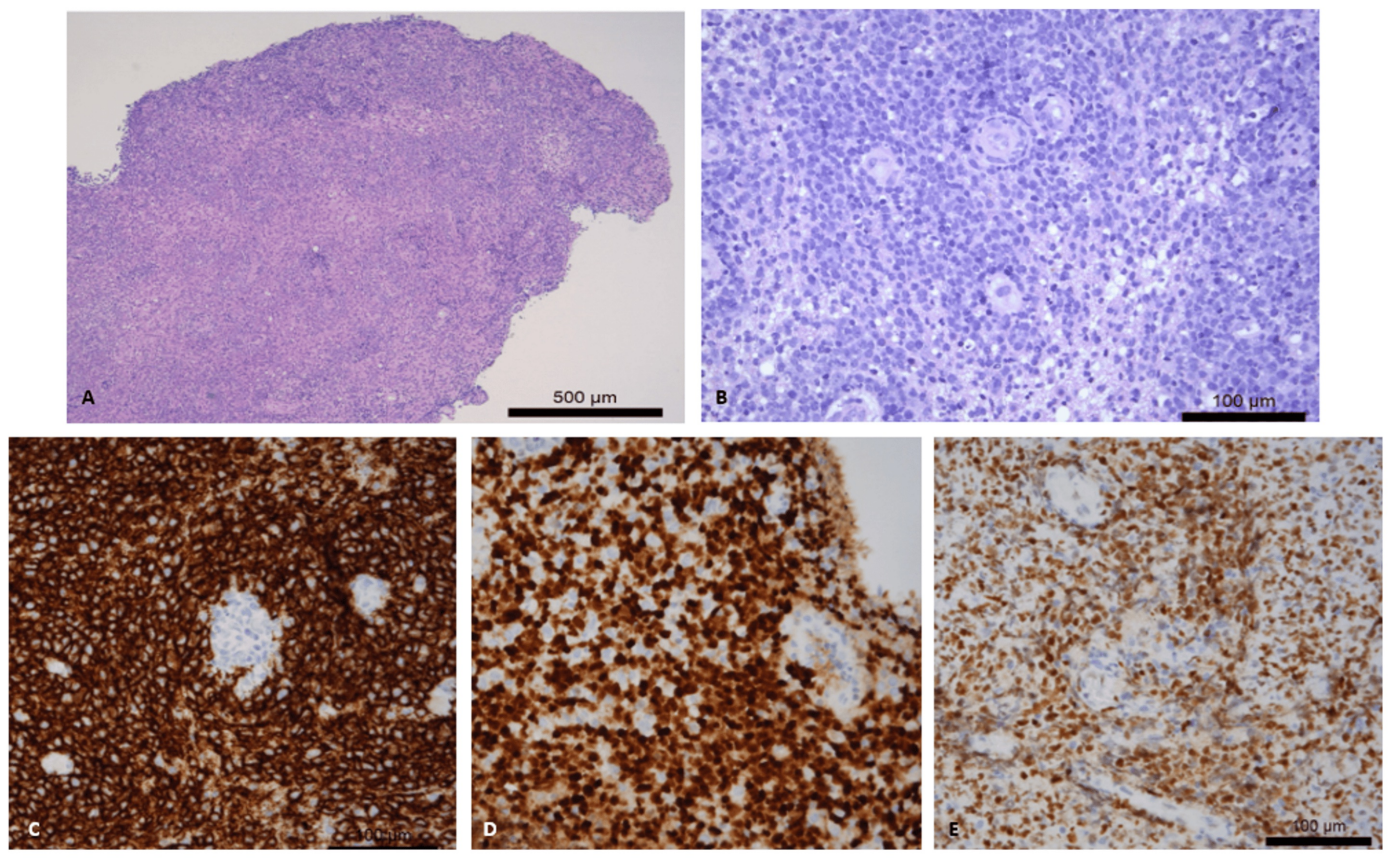
(A, B) H&E: Infiltrative lymphoid neoplasm of the brain parenchyma, with cells showing large nuclei and prominent nucleoli, predominantly T-lymphocytes. (C-E) Immunohistochemistry (CD20, MUM-1, and BCL-6): Neoplastic cells are diffusely positive for CD20 and MUM-1, 80-90% positive for BCL-6, and negative for CD10. H&E, hematoxylin and eosin; CD: cluster of differentiation; MUM-1, multiple myeloma oncogene 1; BCL-6, B-cell lymphoma 6

Twenty months after clinical onset and nine months after corticosteroid treatment, the patient's condition continued to decline, with no definitive diagnosis despite therapy. The case was subsequently discussed at a multidisciplinary meeting involving neurology, neuroradiology, and neuropathology, where it was decided to repeat the cerebromeningeal biopsy, this time targeting the cerebellar lesion. This biopsy confirmed an infiltrative lymphoid neoplasm of the brain parenchyma, with immunohistochemical findings consistent with a diagnosis of diffuse large B-cell lymphoma (Figure 8).

## Discussion

This case highlights the challenge of establishing a definitive diagnosis in a patient with rapidly progressive dementia caused by primary CNS lymphoma. Several factors contributed to this difficulty, including the broad differential diagnosis of RPD, the patient’s immunocompetent status, the immune-related comorbidities that initially biased the diagnostic reasoning, the absence of specific neurological presentations associated with PCNSL, and the limitations of available diagnostic tools.

Initially, the diagnosis of SREAT was considered, given the patient’s clinical presentation and medical history. SREAT has a heterogeneous phenotype, with non-specific clinical and imaging findings. Although it is a rare complication of Hashimoto’s thyroiditis, the patient initially fulfilled the major diagnostic criteria: new-onset cognitive impairment, elevated serum anti-thyroid antibodies (anti-thyroglobulin), and exclusion of infectious, toxic, metabolic, or tumoral etiologies [5].

CSF analysis in SREAT can be normal or show inflammatory changes, with elevated protein levels in 60% of cases [6]. Brain MRI may be normal or reveal non-specific white matter signal changes. About 50% of patients have non-enhancing abnormalities on MRI, with increased T2/FLAIR signal in the white matter and dural enhancement [7]. However, the patient’s lack of clinical response to corticosteroid therapy, a crucial criterion, ultimately argued against this hypothesis.

Autoimmune encephalitis was also considered. The patient’s rapid cognitive decline, presumed epileptic seizures, evidence of inflammatory lesions, and absence of competing causes could meet the Graus criteria for possible seronegative autoimmune encephalitis [8]. However, the progression of cognitive deterioration exceeded the three-month interval defined in the diagnostic criteria, constituting an atypical presentation and prompting investigation of alternative causes. 

PCNSL is rare, with most cases (90-95%) classified histologically as diffuse large B-cell lymphoma [9]. Clinical manifestations depend on the affected brain structures. The cerebral parenchyma is the most frequently involved area, though other structures may also be affected.

In intracranial lesions, cognitive and neuropsychiatric symptoms are among the most common signs, particularly behavioral changes, apathy, and psychomotor slowing, as observed in this patient. Leptomeningeal involvement may present with cranial neuropathies (commonly affecting cranial nerves VI and VII), encephalopathy, seizures, headaches, ataxia, and peripheral neuropathy. Ocular involvement may cause monocular or binocular visual symptoms, including decreased visual acuity, blurred vision, and floaters. Finally, spinal cord involvement typically manifests as subacute, progressive myelopathy [10].

In immunocompetent individuals, lymphoma-related lesions are unifocal in 60-70% of cases and supratentorial in 82% [11]. The most frequently affected regions include the frontal lobes, periventricular white matter, thalamus, basal ganglia, and corpus callosum [12,13]. Less commonly, the infratentorial regions may also be involved, with the cerebellum being the most frequently affected structure. Isolated spinal cord involvement is rare [14].

The imaging pattern of PCNSL is heterogeneous, often mimicking other pathologies such as demyelinating diseases. Lesions are typically hypointense on T1-weighted MRI with contrast enhancement and iso- or hypointense on T2-weighted sequences, showing homogeneous contrast uptake, which may present ring enhancement in up to 13% of cases, along with diffusion restriction [15]. In this patient, the initial lesions did not exhibit these characteristics. These findings may be influenced by the cytotoxic effects of corticosteroid therapy on lymphoma cells, mitigating contrast uptake and metabolic patterns [16]. Non-enhancing lesions tend to be lower grade, less aggressive, and have a better prognosis. The corticospinal tract involvement observed in the patient's second and third MRIs was non-specific but supported the diagnosis. The cerebellar lesion in the fourth MRI, though not in the most characteristic location due to its infratentorial positioning, exhibited diffusion restriction and contrast uptake, supporting the diagnosis of lymphoma [17].

Regarding CSF analysis, typical findings include elevated protein levels (median 235 mg/dL), reflecting significant disruption of the blood-brain barrier, particularly in lymphomas with leptomeningeal involvement. Pleocytosis (median 96/mm³) and low glucose levels (median 47 mg/dL) may also be present [18]. However, protein levels can be normal in 33-55% of patients and cell counts in 33-60%, as seen in this case [3]. In a series of 68 immunocompetent PCNSL patients analyzed, 10% had hypoglycorrhachia [19].

CSF cytology and flow cytometry complement each other by assessing lymphocyte morphology and immunophenotype, potentially providing a definitive diagnosis. Cytology is highly specific; it can identify atypical lymphoid cells, pleomorphic lymphocytes with increased size, irregular nuclear contours, and prominent nucleoli, which are suggestive of lymphoma. However, its sensitivity is low (approximately 2-32%), further reduced after corticosteroid therapy or when small sample volumes are analyzed [20]. Additionally, the morphological similarity between neoplastic and inflammatory lymphocytes can make result interpretation more challenging [3]. Despite these limitations, it can be hypothesized that cytology and flow cytometry, although performed later in this patient, could have contributed to an earlier diagnosis if conducted at an earlier stage, before corticosteroid therapy, taking into account their sensitivity and specificity.

Given the limitations of CSF analysis and MRI, stereotactic biopsy of brain parenchyma is often essential for diagnosing PCNSL. The most common findings include highly proliferative B-cells with an angiocentric growth pattern. Immunohistochemical analysis typically shows positive staining for cluster of differentiation (CD) 19, CD20, B-cell lymphoma 6 (BCL-6) (60-80%), and multiple myeloma oncogene 1 (MUM-1) (80-90%), while CD10, CD38, and CD138 are negative, consistent with findings from the patient's second biopsy [21].

PCNSL cells are sensitive to corticosteroid therapy, which may induce cell cycle arrest, apoptosis, and temporary tumor regression via activation of the p38-mitogen-activated protein kinase (p38-MAPK) pathway [22]. This effect raises the possibility that methylprednisolone followed by prednisolone may have influenced both imaging and histopathological findings. It is known that PCNSL patients previously treated with corticosteroids can exhibit a wide range of histopathological, immunohistochemical, and cytological alterations [23].

Literature reports describe variable effects of corticosteroid therapy on PCNSL diagnosis. In a study of 25 patients who had previously received corticosteroid treatment, 48% achieved a definitive diagnosis based on biopsy, while 52% showed non-specific alterations, some resembling non-neoplastic conditions such as vasculitis and meningoencephalitis, similar to the findings of the patient’s first biopsy. However, in most of these cases, dexamethasone was used at doses of 16 mg/day for a duration ranging from two to 30 days (median of five days), with a short interval (0-2 days) between treatment discontinuation and biopsy [23]. In this case, although a less potent corticosteroid was used (approximately six times weaker) and the interval between treatment cessation and the first biopsy was longer (four months), the biopsy remained non-diagnostic. This suggests that corticosteroid-induced alterations may persist longer than previously reported in the literature. Therefore, in cases of rapidly progressive dementia of unclear etiology, with suspected PCNSL, early biopsy is crucial to avoid corticosteroid-induced histopathological changes and ensure a definitive diagnosis [24].

## Conclusions

PCNSL is a disease with a challenging diagnosis due to its heterogeneous presentation, the wide range of differential diagnoses, and complementary diagnostic tests with low sensitivity. Therefore, a high degree of suspicion is required, and biopsy should be considered in all cases where diagnostic uncertainty exists, given its therapeutic implications. In this case, the role of corticosteroid therapy in delaying diagnosis and the usefulness of repeating brain biopsy when the etiology remains uncertain are emphasized.
